# Arsenicum-album

**Published:** 1896-02

**Authors:** 


### HOMŒOPATHIC PHYSICIAN,

A MONTHLY JOURNAL OF

HOMŒOPATHIC MATERIA MEDICA AND CLINICAL MEDICINE.

"If our school ever gives up the strict inductive method of Hahnemann, we are lost, and deserve only to be mentioned as a caricature in the history of medicine."—CONSTANTINE HERING.

Vol. XVI. FEBRUARY, 1896. No. 2.

### EDITORIAL.

ARSENICUM-ALBUM.—The Arsenic patient has a pulse that is frequent in the morning and slower toward evening. This is especially true of fevers that have been treated with Quinine. The Arsenic patient has fever which is not followed by perspiration. He has fever also from drinking too much wine or other stimulant. This is similar to Silicea. The Arsenic patient is troubled with chilliness or coldness immediately after drinking water. He has heat at night without thirst, and without succeeding perspiration. His thirst is mainly for beer.

If the Arsenicum patient gets perspiration, it comes on at night, is cold, and discontinues during sleep.

Arsenic has chilliness without thirst and heat without thirst.

Aconite has chilliness with thirst and heat with thirst.

Arsenic has heat without inclination to uncover.

Aconite has heat with inclination to uncover.

Arsenicum and Natrum-muriaticum are relieved of various symptoms by the breaking out of perspiration.

Amelioration from perspiration is the key-note of Natrum-muriaticum.

Mercurius has perspiration without relief. This is a key-note.

According to Dr. Hering, Arsenic does not experience relief from perspiration if it occur in the morning.

At all events, there are two symptoms given above that the reader should bear well in mind—Natrum-muriaticum amelioration from perspiration, and Mercurius, *no* amelioration from perspiration. Both of these indications are, as before stated, key-notes.

The Arsenic patient has burning, itching of the skin of the feet, with peeling off of the skin like fish-scales.

Arsenic has general anasarca. It also has petechiæ, similarly to Bryonia and Sulphur.

According to Dr. Guernsey, Arsenic has intertrigo worse in spells.

Arsenic has intense burning pains of carbuncles, worse at night, and with strong desire for alcoholic drinks. This desire must not be gratified, as death is likely to ensue. Arsenic corrects the desire for alcohol under such circumstances. Indeed, Dr. Lippe declared very strongly in his lecture upon Arsenic, that "if the appetite for whisky or brandy occurring in those suffering from carbuncles be indulged, the patient will be a corpse in twenty-four hours." These are his words *verbatim*.

Arsenic has burning like fire around ulcers with offensive odor, and but slight discharge of pus. The ulcers discharge partially coagulated blood. Arsenic is indicated in destructive ulcers of a cancerous nature, easily bleeding.

The Arsenic pains occur when lying down in the evening, and especially after midnight. The pains waken him shortly after midnight, and occur every night. Sometimes they occur every fourth night.

The Arsenic pains are worse from rest and lying down, and better from standing and from motion.

Arsenic is similar to Pulsatilla in having attacks of pain with chilliness.

It is similar to Anacardium and China in its indication for periodical complaints.

Arsenic is indicated in the prostration following the abuse of Quinine.

Arsenic has aggravation on wakening and from cold air. It also has aggravation from becoming cold. Secale has amelioration from getting cold, and Phosphorus has amelioration from cold food. Arsenic has palpitation, weakness, and anguish, worse from cold food.

Arsenic is a great remedy in the bad effects from animal poisons, such as dissecting wounds.

It has aggravation from over-exertion of the body.

As before stated, it has aggravation after midnight. Phosphorus has aggravation before midnight. Calcarea-carbonica has aggravation after three o'clock in the morning.

Arsenic has amelioration from external heat. This is similar to Silicea.

Arsenic vomits food immediately. This is similar to Pulsatilla.

The burning pains of Arsenic also indicate Carbo-vegetabilis, Euphorbium, Mezereum, Pulsatilla, Secale.

The Arsenic patient is sensitive to cold even in warm weather. The pains are relieved most by motion. There is nausea and anguish with extreme prostration. The complaints are periodical, and worse during rest.

Here are two or three of the key-notes of Arsenic as given by the late Dr. Henry N. Guernsey:

Thirst for cold water; very little satisfies, but it is required very often. Unhappy, fatiguing dreams; nothing comes out right in the dreams. Erectile tumors, with burning, lancinating pains. Varicose veins, that burn like fire.

Many more of these key-notes of Dr. Guernsey might be added. The editor has in his possession a manuscript book containing a complete collection of the key-notes of Dr. Guernsey just as they stood in his *Obstetrics*, with a few additions from his lectures. This book was strongly and neatly bound, and is in daily use in the treatment of cases. The editor would be glad to publish this book in full, with the addition of a repertory, and, indeed, has been urged to do so by a distinguished homœopathist residing on the Pacific coast. Unfortunately, a copyright upon a previous publication by Dr. Guernsey's son having this same title, but arranged on a different plan, stands in the way, and so it is not possible to do so at the present time.

